# First person – Anna Gray

**DOI:** 10.1242/dmm.044875

**Published:** 2020-05-26

## Abstract

First Person is a series of interviews with the first authors of a selection of papers published in Disease Models & Mechanisms (DMM), helping early-career researchers promote themselves alongside their papers. Anna Gray is first author on ‘ Deterioration of muscle force and contractile characteristics are early pathological events in spinal and bulbar muscular atrophy mice’, published in DMM. Anna conducted the research described in this article while a PhD student in Prof. Linda Greensmith's lab at the Department of Neuromuscular Disease, UCL Institute of Neurology, Queen Square, London, UK. She is now a postdoctoral research associate in systems neurobiology in the lab of Prof. Rasmus Petersen at the Division of Neuroscience & Experimental Psychology, Faculty of Biology, Medicine & Health, University of Manchester, UK, investigating the development of reliable *in vivo* techniques to study neurobiology in health and disease.


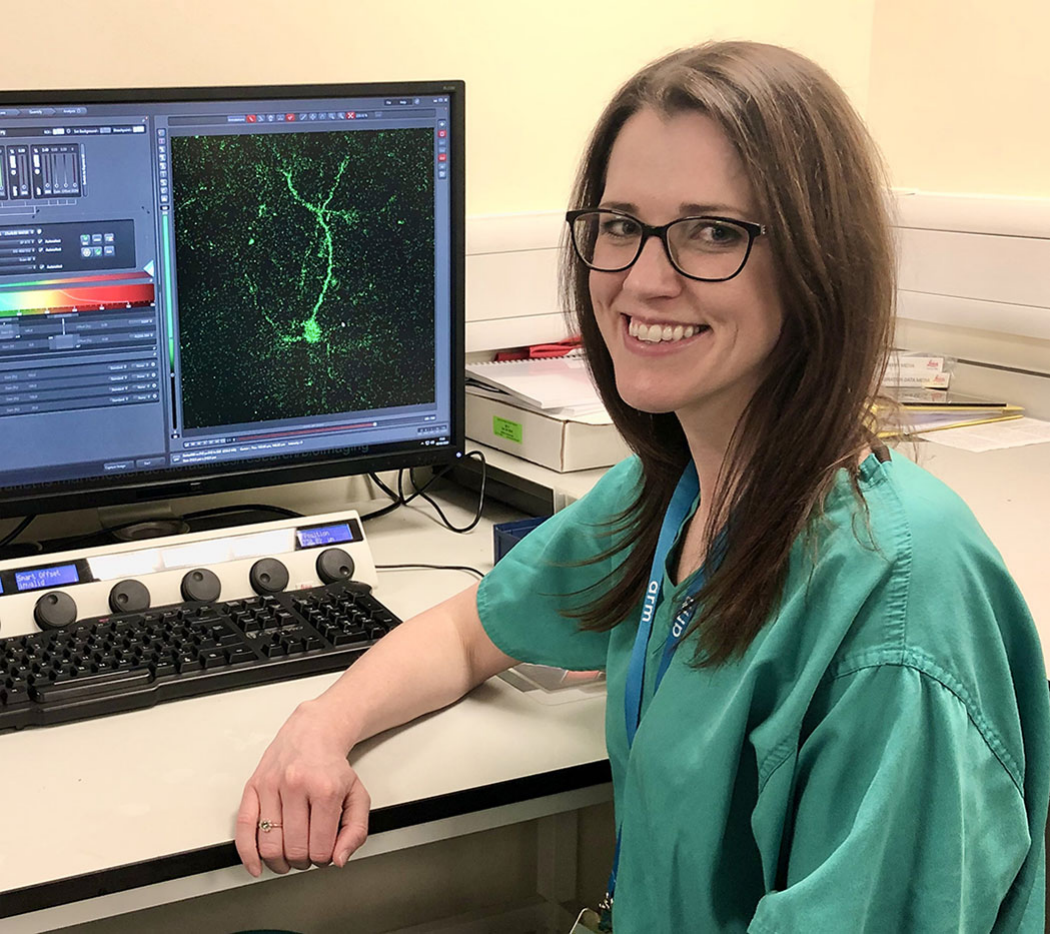


**Anna Gray**

**How would you explain the main findings of your paper to non-scientific family and friends?**

Spinal and bulbar muscular atrophy (SBMA), also known as Kennedy's disease, is a neuromuscular disease characterised by loss of motor neurons in the spinal cord, which control muscle function, with accompanying muscle weakness and wasting. There are currently no effective treatments for this debilitating disease. In this study we used a mouse model of SBMA and conducted a comprehensive analysis of muscle function and neurological deficits through the disease course, in order to identify key stages. This is especially important in disorders such as SBMA, where disease can be identified before symptom onset, through family history and genetic testing. Thus, treatment can be initiated early. Our results show that muscle is the primary site of disease pathology and that the nervous system is only affected in late-stage disease. Importantly, these findings indicate that targeting muscle deficits may be an effective therapeutic strategy in SBMA. This is favourable as muscle is a more accessible therapeutic target than the central nervous system (CNS).

**What are the potential implications of these results for your field of research?**

SBMA is a genetic disorder caused by a trinucleotide repeat expansion in the gene that encodes the androgen receptor (AR). Thus, patients can be identified through genetic testing and family history. Our results show that in the AR100 mouse model of SBMA, despite generally being considered a motor neuron disease, symptoms first manifest in hindlimb skeletal muscle, before any motor neuron degeneration, which only occurs in late-stage disease. These findings confirm that muscle plays a key role in disease pathogenesis in SBMA and, indeed, may be the primary site of AR toxicity, suggesting that muscle-targeted therapeutics may be particularly effective. Importantly, peripheral tissues such as muscle present an attractive target for therapy, as they are more accessible than CNS targets. Treatment may be initiated before disease manifestation.

**What are the main advantages and drawbacks of the model system you have used as it relates to the disease you are investigating?**

AR100 mice closely recapitulate the slowly progressive neuromuscular phenotype exhibited in human SBMA patients. Importantly, the pathology seen in this model appears to be restricted to the lower motor neuron degeneration and muscle pathology seen in patients, thus maintaining the cell-type specificity of the human disease, unlike other AR transgenic mouse models. In later disease stages many AR100 mice have a significant decline in the ability to ambulate, just as many SBMA patients can become wheelchair bound. Disease is caused by a trinucleotide repeat expansion in the gene that encodes AR, positioned on the X chromosome. Therefore, SBMA predominantly affects males, and this strong gender effect is maintained in AR100 mice. Although SBMA is a debilitating disease, it generally does not affect life expectancy in humans; likewise AR100 mice recapitulate disease course over the lifespan of the mouse, reaching designated end stage of disease at 18 months. All things considered, AR100 mice make an ideal model to evaluate the natural history of the disease alongside effectiveness of therapeutics. The trinucleotide repeat expansion required to generate disease in AR100 mice is significantly greater than that seen in patients. However, this may be because of the fact that endogenous, non-mutant AR is still present in these animals and may rescue the disease phenotype to some extent. Owing to the slowly progressive nature of disease in AR100 mice, the analysis of disease progression and response to therapeutic intervention is considerably time consuming.

**Figure:**
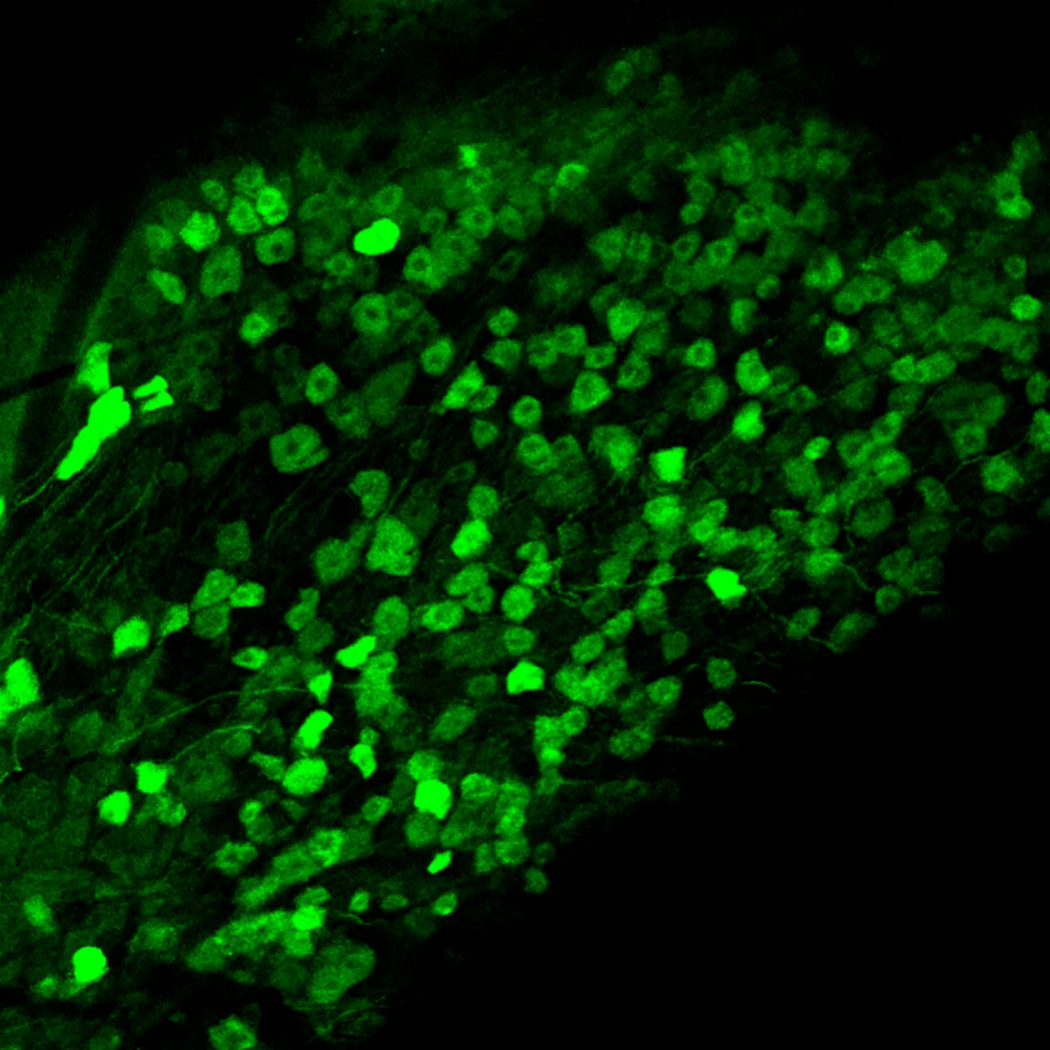
**Maximum projection of a confocal *xyz* anatomical stack showing GCaMP6-expressing neurons in the mouse trigeminal ganglion.**

**What has surprised you the most while conducting your research?**

While conducting the research documented in this paper I was very impressed by the sensitivity and reliability of the muscle physiology experiments used. The muscle force and contractile characteristics were very reproducible between all animals at each of the designated time points, and the error bars for this data are considerably small. These tests were also able to distinguish muscle deficits before presentation of any symptoms. I was surprised to identify muscle deficits at such an early disease stage (6 months). Weight differences between wild-type and AR100 mice become very apparent through the disease course. However, we have shown that muscle deficits precede any disease-related decline in bodyweight in SBMA mice.

“[…] I was very impressed by the sensitivity and reliability of the muscle physiology experiments used.”

**Describe what you think is the most significant challenge impacting your research at this time and how will this be addressed over the next 10 years?**

I am currently using a Thy1-GCaMP6 transgenic mouse line to analyse calcium dynamics in the trigeminal ganglion in response to controlled stimuli. My biggest challenge at present is the sparse GCaMP6 expression within neurons. This makes it difficult to understand how representative the responding cells in our experiments are in the context of the neuronal population as a whole. In the coming years it would be good to develop a new mouse model using a different promoter, enabling *in vivo* recording from a larger proportion of the neuronal population.

**What changes do you think could improve the professional lives of early-career scientists?**

Our University provides many core facilities, which provide expertise and training for many of the techniques that we may utilise in our research. However, I feel that it would be beneficial to have more guidance in terms of how to promote ourselves as early-career researchers. Furthermore, it would be good to get support in this context from senior colleagues by ensuring our inclusion on lab web pages and the detailing of our research.

**What's next for you?**

I have now finished my PhD and am several years into my postdoctoral career. My current research encompasses live intravital microscopy, requiring intricate surgical methodologies, alongside confocal and multi-photon imaging. My initial endeavours focused on the implantation of cranial windows in transgenic Thy1-GCaMP6 mice and multiphoton imaging of calcium dynamics in the mouse cortex. However, in an initiative to explore novel imaging targets, I have established a decerebration model to expose the trigeminal ganglion at the base of the skull. This has not only enabled calcium imaging of this structure at the population level for the first time, but also allowed investigation into the response to certain stimulus paradigms. My objective now is to use this live *in vivo* imaging setup to study disease models and the effectiveness of potential therapeutics.
